# Supplementary material for: Monocyte distribution width enhances the detection of infection in patients after primary percutaneous coronary intervention
Source: PLoS One. 2025 Jun 3;20(6):e0325314. doi: 10.1371/journal.pone.0325314 (PMC12132996; doi:10.1371/journal.pone.0325314)
Supplement: S1 File — S1 Table. Characteristics of patients with acute myocardial infarction and newly diagnosed infections. S2 Table. Diagnostic performance of predictors for newly diagnosed infections. S3 Table. Univariable analysis results of length of stay ≥ 7 days. S4 Table. Diagnostic performance of the prediction of length of stay ≥ 7 days. S5 Table. Multivariable analysis results of length of stay ≥ 7 days (including the sensitivity analysis by replacing qSOFA with SIRS score). S6 Table. Comparisons for model for predicting newly diagnosed infection. S7 Table. Sensitivity analysis: multivariable analysis for prediction of new infection in patients with acute coronary syndrome (N = 252). S8 Table. Sensitivity analysis: comparisons for model for predicting newly diagnosed infection. S9 Table. Subgroup analysis for the results of newly diagnosed infection (N = 252). S1 Fig. The study flow diagram. N = 310 for acute coronary syndrome patients were screened for enrollment. S2 Fig. Timing of newly diagnosed infections during hospitalization. N = 32 for acute coronary syndrome patients treated after PCI with infection. S3 Fig. Distribution of Monocyte Distribution Width (MDW) by Infection Onset. This box-and-whisker plot displays the distribution of MDW values across different days of infection onset, with individual data points overlaid as jittered dots. Notably, the median MDW on day 0—the day of newly diagnosed infection—was above 20, and higher than that observed on subsequent days (days 1 and 2). This finding supports our predictive model, which identified MDW ≥ 20 as a significant threshold for newly diagnosed infection, reinforcing MDW’s role as an early biomarker in acute infection settings. S4 Fig. Diagnostic performance of simplified models with three parameters (a) and four parameters (b). While the three-parameter models included age ≥ 65 years, MDW ≥ 20, and SIRS ≥ 2, the four-parameter models included the same three parameters in addition to CRP ≥ 2 mg/dL. S5 Fig. Sensitiv [file pone.0325314.s001.docx]

**Supplemental Material**

**List of Supplemental Tables**

**S1 Table. Characteristics of Patients With Acute Myocardial Infarction and Newly Diagnosed Infections.**

**S2 Table. Diagnostic Performance of Predictors for Newly Diagnosed Infections.**

**S3 Table. Univariable Analysis Results of Length of Stay ≥ 7 Days.**

**S4 Table. Diagnostic Performance of the Prediction of Length of Stay ≥ 7 Days**

**S5 Table. Multivariable Analysis Results of Length of Stay ≥ 7 Days (Including the Sensitivity Analysis by Replacing qSOFA with SIRS score).**

**S6 Table. Comparisons for Model for Predicting Newly Diagnosed Infection.**

**S7 Table. Sensitivity Analysis: Multivariable Analysis for Prediction of New Infection in Patients with Acute Coronary Syndrome (*N* = 252).**

**S8 Table. Sensitivity Analysis: Comparisons for Model for Predicting Newly Diagnosed Infection**

**S9 Table. Subgroup Analysis for the Results of Newly Diagnosed Infection (*N* = 252).**

**List of Supplemental Figures**

**S1 Fig. The study flow diagram.**

**S2 Fig. Timing of newly diagnosed infections during hospitalization.**

**S3 Fig. Distribution of Monocyte Distribution Width (MDW) by Infection Onset.**

**S4 Fig. Diagnostic performance of simplified models with three parameters (a) and four parameters (b).**

**S5 Fig. Sensitivity analysis: comparison of the diagnostic performance of the C-ACS and SHR models with our models for the prediction of infection during hospitalization.**

**S1 Table.** Characteristics of Patients With Acute Myocardial Infarction and Newly Diagnosed Infections

| Patient no. | Time of antibiotic administration | Time of fever | Total length of stay (days) | Site of infection | Sepsis | MDW |
| --- | --- | --- | --- | --- | --- | --- |
| 1 | Day 3 | No fever | 4 | UTI | Absent | 19.9 |
| 2 | Day 0 | Day 0 | 14 | UTI | Absent | 22.4 |
| 3 | Day 9 | Day 8 | 15 | UTI | Absent | 20.8 |
| 4 | Day 2 | Day 2 | 21 | UTI | Absent | 20.5 |
| 5 | Day 1 | Day 1 | 83 | UTI | Present | 21.5 |
| 6 | Day 3 | Day 3 | 12 | UTI | Absent | 17.0 |
| 7 | Day 0 | No fever | 22 | Pneumonia | Present | 23.2 |
| 8 | Day 0 | Day 7 | 18 | Pneumonia | Present | 16.5 |
| 9 | Day 0 | Day 4 | 7 | Pneumonia | Absent | 21.8 |
| 10 | Day 1 | No fever | 6 | Pneumonia | Absent | 17.7 |
| 11 | Day 2 | No fever | 11 | Pneumonia | Absent | 22.3 |
| 12 | Day 0 | No fever | 8 | Pneumonia | Absent | 21.8 |
| 13 | Day 0 | No fever | 18 | Pneumonia | Absent | 22.2 |
| 14 | Day 0 | No fever | 16 | Pneumonia | Absent | 18.2 |
| 15 | Day 0 | Day 0 | 20 | Pneumonia | Present | 25.0 |
| 16 | Day 0 | No fever | 10 | Pneumonia | Absent | 23.2 |
| 17 | Day 0 | Day 5 | 73 | Pneumonia | Present | 15.1 |
| 18 | Day 0 | Day 0 | 9 | Pneumonia | Absent | 23.8 |
| 19 | Day 0 | Day 4 | 45 | Pneumonia | Present | 25.5 |
| 20 | Day 0 | No fever | 12 | Pneumonia | Absent | 24.3 |
| 21 | Day 0 | Day 0 | 10 | Pneumonia | Present | 28.8 |
| 22 | Day 0 | No fever | 14 | Pneumonia | Present | 19.2 |
| 23 | Day 0 | No fever | 24 | Pneumonia | Present | 21.0 |
| 24 | Day 2 | Day 2 | 36 | Pneumonia | Present | 16.8 |
| 25 | Day 0 | Day 8 | 26 | Pneumonia | Present | 18.8 |
| 26 | Day 0 | Day 5 | 25 | Pneumonia | Absent | 14.3 |
| 27 | Day 0 | Day 2 | 11 | Pneumonia | Absent | 15.2 |
| 28 | Day 0 | Day 1 | 44 | Pneumonia | Present | 17.6 |
| 29 | Day 0 | Day 2 | 40 | Pneumonia | Absent | 28.2 |
| 30 | Day 0 | Day 3 | 25 | Pneumonia | Present | 19.6 |
| 31 | Day 3 | No fever | 36 | IAI | Present | 24.9 |
| 32 | Day 5 | No fever | 17 | IAI | Absent | 18.0 |

Abbreviations: IAI, intra-abdominal infection; MDW, monocyte distribution width; UTI, urinary tract infection.

**S2 Table 2.** Diagnostic Performance of Predictors for Newly Diagnosed Infections

| Characteristics  (*N* = 252) | AUC (95% CI) | Sensitivity (95% CI) | Specificity (95% CI) |
| --- | --- | --- | --- |
| Age (years) | 0.744 (0.645–0.843) | — | — |
| Age ≥65 years | 0.680 (0.597–0.762) | 75.0% (60.0%–90.0%) | 60.9% (54.5%–67.4%) |
| Body temperature (°C) | 0.523 (0.391–0.655) | — | — |
| Heart rate (beats/min) | 0.630 (0.513–0.747) | — | — |
| Heart rate ≥ 90 beats/min | 0.610 (0.519–0.701) | 37.5% (20.7%–54.3%) | 40.5% (34.0%–46.9%) |
| Respiratory rate (breaths/min) | 0.663 (0.544–0.782) | — | — |
| Respiratory rate ≥20 breaths/min | 0.686 (0.595–0.776) | 40.6% (23.6%–57.6%) | 22.3% (16.8%–27.8%) |
| SBP (mmHg) | 0.555 (0.439–0.672) | — | — |
| DBP (mmHg) | 0.605 (0.493–0.717) | — | — |
| MAP (mmHg) | 0.586 (0.473–0.699) | — | — |
| SIRS score (per one unit of increase) | 0.691 (0.584–0.798) | — | — |
| SIRS score ≥ 2 | 0.672 (0.581–0.764) | 46.9% (29.6%–64.2%) | 18.6% (13.5%–23.8%) |
| qSOFA score (per one unit of increase) | 0.712 (0.619–0.806) |  |  |
| qSOFA score ≥ 2 | 0.594 (0.520–0.667) | 78.1% (63.8%–92.5%) | 3.2% (0.9%–5.5%) |
| Medical comorbidity |  |  |  |
| Hypertension | 0.529 (0.436–0.622) | — | — |
| Diabetes mellitus | 0.513 (0.426–0.600) | — | — |
| Prior CAD | 0.531 (0.440–0.621) | — | — |
| Previous stroke | 0.524 (0.481–0.568) |  |  |
| Malignancy | 0.527 (0.484–0.570) |  |  |
| CK | 0.536 (0.424–0.648) |  |  |
| CK-MB | 0.606 (0.496–0.716) |  |  |
| Troponin-T | 0.401 (0.303–0.499) |  |  |
| MDW | 0.763 (0.651–0.876) | — | — |
| MDW ≥ 20 | 0.731 (0.642–0.821) | 56.3% (39.1%–73.4%) | 90.0% (86.0%–94.0%) |
| WBC (10^3^ cells/μL) | 0.693 (0.599–0.786) | — | — |
| WBC ≥ 10500 cells/μL | 0.617 (0.529–0.705) |  |  |
| CRP | 0.733 (0.618–0.848) | — | — |
| CRP ≥ 2 mg/dL | 0.758 (0.670–0.846) | 37.5% (20.7%–54.3%) | 10.9% (6.8%–15.0%) |
| NLR | 0.643 (0.518–0.769) | — | — |
| NLR ≥ 4 | 0.614 (0.526–0.702) | 31.3% (15.2%–47.3%) | 45.9% (39.3%–52.5%) |

Abbreviations: AUC; area under the curve; CAD, coronary artery disease; CK, creatine kinase; CRP, C-reactive protein; DBP, diastolic blood pressure; ED, emergency department; SBP, systolic blood pressure; SIRS, systemic inflammatory response syndrome; MAP, mean arterial pressure; MDW, monocyte distribution width; NLR, neutrophil-to-lymphocyte ratio; qSOFA, Quick Sequential Organ Failure Assessment.

^*^Statistically significant (*P* < 0.05).

^†^CRP levels were measured in only 107 patients in the ED.

**S3 Table 3.** Univariable Analysis Results of Length of Stay ≥ 7 Days

| Characteristics  (*N* = 252) | OR (95% CI) | *P* value |
| --- | --- | --- |
| Age (years) | 1.05 (1.03–1.08) | <0.0001^*^ |
| Age ≥65 years | 3.24 (1.82–5.76) | <0.0001^*^ |
| Sex (male vs. female) | 0.64 (0.33–1.25) | 0.1911 |
| BMI (kg/m^2^) | 0.95 (0.86–1.04) | 0.2844 |
| Chest pain | 5.78 (3.02–11.06) | <0.0001^*^ |
| Dyspnea | 5.73 (2.95–11.14) | <0.0001^*^ |
| Syncope | 2.17 (0.90–5.20) | 0.0833 |
| Body temperature (°C) | 1.16 (0.67–2.00) | 0.5987 |
| Heart rate (beats/min) | 1.02 (1.01–1.03) | 0.0017^*^ |
| Heart rate ≥ 90 beats/min | 2.58 (1.46–4.53) | 0.0010^*^ |
| Respiratory rate (breaths/min) | 1.20 (1.09–1.31) | 0.0002^*^ |
| Respiratory rate ≥20 breaths/min | 3.42 (1.88–6.19) | <0.0001^*^ |
| SBP (mmHg) | 1.00 (0.99–1.01) | 0.4207 |
| DBP (mmHg) | 0.99 (0.98–1.00) | 0.0725 |
| MAP (mmHg) | 0.99 (0.98–1.00) | 0.1559 |
| SIRS score (per score increase) | 2.15 (1.56–2.97) | <0.0001^*^ |
| SIRS score ≥ 2 | 4.13 (2.22–7.68) | 0.0003^*^ |
| qSOFA score (per score of increase) | 4.00 (2.40–6.65) | <0.0001^*^ |
| qSOFA score ≥ 2 | 7.13 (2.28–22.24) | 0.0007^*^ |
| Infection | 67.50 (15.49–294.10) | <0.0001^*^ |
| Sepsis | 93.81 (4.98–999.99) | 0.0024^*^ |
| Hypertension | 1.51 (0.87–2.63) | 0.1472 |
| Diabetes mellitus | 1.07 (0.59–1.96) | 0.8228 |
| Prior CAD | 1.77 (0.99–3.14) | 0.0516 |
| Previous stroke | 1.76 (0.29–10.74) | 0.5425 |
| Malignancy | 2.65 (0.37–19.16) | 0.3355 |
| CK | 1.00 (1.00–1.00) | 0.6974 |
| CK-MB | 1.00 (1.00–1.00) | 0.9219 |
| Troponin-T | 0.99 (0.84–1.17) | 0.9173 |
| MDW | 1.31 (1.16–1.49) | <0.0001^*^ |
| MDW ≥ 18 | 2.55 (1.45–4.49) | 0.0012^*^ |
| MDW ≥ 20 | 4.18 (2.07–8.42) | <0.0001^*^ |
| WBC (10^3^ cells/μL) | 1.08 (1.01–1.16) | 0.0332^*^ |
| WBC ≥ 10500 cells/μL | 1.63 (0.93–2.84) | 0.0867 |
| CRP | 1.15 (1.04–1.28) | 0.0059^*^ |
| CRP ≥ 2 mg/dL | 8.16 (3.89–17.13) | <0.0001^*^ |
| NLR | 1.10 (1.03–1.18) | 0.0038^*^ |
| NLR ≥ 4 | 2.14 (1.22–3.74) | 0.0078^*^ |

Abbreviations: CAD, coronary artery disease; CRP, C-reactive protein; DBP, diastolic blood pressure; ED, emergency department; PCT, procalcitonin; SBP, systolic blood pressure; SIRS, systemic inflammatory response syndrome; SpO_2_, peripheral capillary oxygen saturation; MAP, mean arterial pressure; OR, odds ratio.

^*^Statistically significant (*P* < 0.05).

**S4 Table 4.** Diagnostic Performance of the Prediction of Length of Stay ≥ 7 Days

| Characteristics  (*N* = 252) | AUC (95% CI) | Sensitivity (95% CI) | Specificity (95% CI) |
| --- | --- | --- | --- |
| Age (years) | 0.681 (0.605–0.758) | — | — |
| Age ≥65 years | 0.643 (0.576–0.709) | 64.3% (53.1%–75.5%) | 64.3% (57.3%–71.3%) |
| Heart rate (beats/min) | 0.631 (0.548–0.714) | — | — |
| Heart rate ≥ 90 beats/min | 0.616 (0.548–0.684) | 60.0% (48.5%–71.5%) | 63.2% (56.2%–70.2%) |
| Respiratory rate (breaths/min) | 0.617 (0.531–0.702) | — | — |
| Respiratory rate ≥20 breaths/min | 0.630 (0.564–0.695) | 45.7% (34.0%–57.4%) | 80.2% (74.4%–86.0%) |
| SIRS score (per one unit of increase) | 0.678 (0.605–0.750) | — | — |
| SIRS score ≥ 2 | 0.637 (0.573–0.701) | 42.9% (31.3%–54.5%) | 84.6% (79.4%–89.9%) |
| qSOFA score (per one unit of increase) | 0.699 (0.635–0.764) |  |  |
| qSOFA score ≥ 2 | 0.566 (0.517–0.615) | 12.9% (5.0%–20.7%) | 97.3% (94.9%–99.6%) |
| MDW | 0.650 (0.567–0.732) | — | — |
| MDW ≥ 18 | 0.615 (0.547–0.682) | 61.4% (50.0%–72.8%) | 61.5% (54.5%–68.6%) |
| MDW ≥ 20 | 0.608 (0.549–0.667) | 31.4% (20.6%–42.3%) | 90.1% (85.8%–94.5%) |
| CRP | 0.698 (0.597–0.799) | — | — |
| CRP ≥ 2 mg/dL | 0.657 (0.597–0.718) | 38.6% (27.2%–50.0%) | 92.9% (89.1%–96.6%) |
| NLR | 0.573 (0.487–0.659) | — | — |
| NLR ≥ 4 | 0.593 (0.525–0.661) | 54.3% (42.6%–66.0%) | 64.3% (57.3%–71.3%) |

Abbreviations: AUC; area under the curve; CI, confidence interval; CRP, C-reactive protein; MDW, monocyte distribution width; NLR, neutrophil-to-lymphocyte ratio; qSOFA, Quick Sequential Organ Failure Assessment; SIRS, systemic inflammatory response syndrome.

**S5 Table.** Multivariable Analysis Results of Length of Stay ≥ 7 Days (Including the Sensitivity Analysis by Replacing qSOFA with SIRS score)

| Characteristics  (*N* = 252) | Model based on qSOFA score  OR (95% CI) | *P* value | Model based on SIRS score  OR (95% CI) | | *P* value |  |
| --- | --- | --- | --- | --- | --- | --- |
| **Full multivariable model** | |  | |  |  |  |
| SIRS score ≥ 2 | — | — | 2.43 (0.90–6.55) | | 0.0793 |  |
| qSOFA score ≥ 2 | 2.07 (0.53–8.03) | 0.2924 | — | | — |  |
| Age ≥65 years | 3.55 (1.77–7.11) | 0.0004^*^ | 3.94 (1.95–7.99) | | 0.0001^*^ |  |
| Heart rate ≥ 90 beats/min | 1.51 (0.77–2.96) | 0.2281 | 0.97 (0.41–2.26) | | 0.9338 |  |
| Respiratory rate ≥20 breaths/min | 2.42 (1.18–4.99) | 0.0162^*^ | 2.22 (1.07–4.64) | | 0.0334^*^ |  |
| MDW ≥ 20 | 2.21 (0.92–5.28) | 0.0754 | 2.41 (1.01–5.75) | | 0.0480^*^ |  |
| CRP ≥ 2 mg/dL | 7.44 (3.13–17.64) | <0.0001^*^ | 6.71 (2.80–16.10) | | <0.0001^*^ |  |
| NLR ≥ 4 | 1.01 (0.51–2.01) | 0.9748 | 0.98 (0.50–1.96) | | 0.9619 |  |
| Model fit |  |  |  | |  |  |
| AUC (95% CI) | 0.798 (0.730–0.865) |  | 0.798 (0.727–0.868) | |  |  |
| AIC | 227.72 |  | 241.69 | |  |  |
| Hosmer–Lemeshow test | 3.517 (9 groups) | 0.8334 | 8.689 (9 groups) | | 0.2758 |  |
| **Simplified multivariable model** | |  | |  |  |  |
| SIRS score ≥ 2 | — | — | 4.22 (2.12–8.44) | | <0.0001^*^ |  |
| qSOFA score ≥ 2 | 3.29 (0.93–11.68) | 0.0658 | — | | — |  |
| NEWS | — | — | — | | — |  |
| Age ≥65 years | 3.10 (1.70–5.67) | 0.0002^*^ | 3.72 (1.98–7.01) | | <0.0001^*^ |  |
| MDW ≥ 20 | 3.93 (1.85–8.33) | 0.0004^*^ | 3.60 (1.65–7.87) | | 0.0013^*^ |  |
| Model fit |  |  |  | |  |  |
| AUC (95% CI) | 0.714 (0.645–0.783) |  | 0.740 (0.670–0.811) | |  |  |
| AIC | 140.97 |  | 255.14 | |  |  |
| Hosmer–Lemeshow test | 0.343 (4 groups) | 0.8425 | 2.965 (5 groups) | | 0.3970 |  |

Abbreviations: AUC, area under the curve; BT, body temperature; CRP, C-reactive protein; NLR, neutrophil-to-lymphocyte ratio; OR, odds ratio; RR, respiratory rate; SIRS, systemic inflammatory response syndrome.

^*^Statistically significant (*P* < 0.05).

**S6 Table.** Comparisons for Model for Predicting Newly Diagnosed Infection

| Model | AUC (95% CI) | IDI (95% CI) | *P* value |
| --- | --- | --- | --- |
| C-ACS | 0.807 (0.732–0.881) | (reference) | — |
| Four-parameters model | 0.909 (0.860–0.958) | 0.819 (0.790–0.847) | <0.0001^*^ |
| Three parameters model | 0.827 (0.751–0.902) | 0.802 (0.775–0.829) | <0.0001 |
| SHR | 0.784 (0.685–0.882) | (reference) | — |
| Four-parameters model | 0.909 (0.860–0.958) | 0.808 (0.780–0.836) | <0.0001^*^ |
| Three parameters model | 0.827 (0.751–0.902) | 0.796 (0.769–0.824) | <0.0001^*^ |

Abbreviations: AUC, area under the curve; C-ACS, Canada Acute Coronary Syndrome; CI, confidence interval; IDI, integrated discrimination improvement.

Integrated discrimination improvement test was used to compare different models and obtain *P* values.

^*^Statistically significant (*P* < 0.0125). To account for multiple comparisons, we applied a Bonferroni correction, adjusting the significance threshold to 0.0125 (α = 0.05 divided by four comparisons). The comparisons remained statistically significant under this correction.

**S7 Table.** Sensitivity Analysis: Multivariable Analysis for Prediction of New Infection in Patients with Acute Coronary Syndrome (*N* = 252)

|  | Full multivariable model | | Four-parameter model | | Three-parameter model | |
| --- | --- | --- | --- | --- | --- | --- |
| Characteristics | OR (95% CI) | *P* value | OR (95% CI) | *P* value | OR (95% CI) | *P* value |
| SIRS score ≥ 2 | 2.74 (0.41–18.44) | 0.3015 | 2.87 (1.04–7.90) | 0.0419^*^ | 4.45 (1.78–11.16) | 0.0015^*^ |
| Age ≥65 years | 7.70 (2.32–25.58) | 0.0009^*^ | 9.50 (3.00–30.04) | 0.0001^*^ | 5.83 (2.20–15.46) | 0.0004^*^ |
| MDW ≥ 20 | 8.29 (2.75–24.97) | 0.0002^*^ | 8.50 (3.03–23.88) | <0.0001^*^ | 11.08 (4.33–28.38) | <0.0001^*^ |
| CRP ≥ 2 mg/dL^†^ | 14.28 (4.16–49.04) | <0.0001^*^ | 11.12 (3.87–31.94) | <0.0001^*^ |  |  |
| Heart rate ≥ 90 beats/min | 0.51 (0.11–2.46) | 0.4050 |  |  |  |  |
| Respiratory rate ≥20/min | 2.76 (0.91–8.33) | 0.0717 |  |  |  |  |
| NLR ≥ 4 | 0.64 (0.21–1.96) | 0.4313 |  |  |  |  |
| WBC ≥ 10500 cells/μL | 1.04 (0.28–3.94) | 0.9500 |  |  |  |  |
| Model fit |  |  |  |  |  |  |
| AUC (95% CI) | 0.909 (0.847–0.970) |  | 0.907 (0.848–0.967) |  | 0.834 (0.752–0.916) |  |
| AIC | 126.32 |  | 121.53 |  | 141.40 |  |
| Hosmer–Lemeshow test | 4.40 (df = 8) | 0.8191 | 1.776 (df = 4) | 0.7768 | 1.692 (df = 3) | 0.6388 |

Abbreviations: AUC, area under the curve; BT, body temperature; CRP, C-reactive protein; df, degrees of freedom; NLR, neutrophil-to-lymphocyte ratio; OR, odds ratio; RR, respiratory rate; SIRS, systemic inflammatory response syndrome.

^*^Statistically significant (*P* < 0.05).

^†^CRP levels were measured in only 107 patients in the ED.

**S8 Table.** Sensitivity Analysis: Comparisons for Model for Predicting Newly Diagnosed Infection

| Model | AUC (95% CI) | IDI (95% CI) | *P* value |
| --- | --- | --- | --- |
| C-ACS | 0.807 (0.732–0.881) | (reference) | — |
| Four-parameter model | 0.907 (0.848–0.967) | 0.820 (0.792–0.847) | <0.0001^*^ |
| Three-parameter model | 0.834 (0.752–0.916) | 0.807 (0.781–0.834) | <0.0001^*^ |
| SHR | 0.784 (0.685–0.882) | (reference) | — |
| Four-parameter model | 0.907 (0.848–0.967) | 0.808 (0.781–0.835) | <0.0001^*^ |
| Three-parameter model | 0.834 (0.752–0.916) | 0.798 (0.771–0.825) | <0.0001^*^ |

Abbreviations: AUC, area under the curve; C-ACS, Canada Acute Coronary Syndrome; CI, confidence interval; IDI, integrated discrimination improvement; SHR, .

^*^Statistically significant (*P* < 0.05).

**S9 Table.** Subgroup Analysis for the Results of Newly Diagnosed Infection (*N* = 252)

|  | Full multivariable model | | Four-parameter model | | Three-parameter model | |
| --- | --- | --- | --- | --- | --- | --- |
| Characteristics | OR (95% CI) | *P* value | OR (95% CI) | *P* value | OR (95% CI) | *P* value |
| STEMI (n=114) | |  |  |  |  |  |
| qSOFA score ≥ 2 | 2.44 (0.32–18.36) | 0.3863 | 3.22 (0.53–19.63) | 0.2045 | 5.23 (1.04–26.40) | 0.0452^*^ |
| Age ≥65 years | 8.87 (1.52–51.74) | 0.0153^*^ | 7.08 (1.42–35.20) | 0.0168^*^ | 3.50 (0.99–12.43) | 0.0524 |
| MDW ≥ 20 | 1.99 (0.27–14.57) | 0.4982 | 2.39 (0.37–15.47) | 0.3620 | 1.82 (0.32–10.34) | 0.4988 |
| CRP ≥ 2 mg/dL | 20.99 (4.02–109.66) | 0.0003^*^ | 22.76 (4.83–107.16) | <0.0001^*^ |  |  |
| Heart rate ≥ 90 beats/min | 1.43 (0.27–7.49) | 0.6742 |  |  |  |  |
| Respiratory rate ≥20/min | 1.94 (0.37–10.08) | 0.4317 |  |  |  |  |
| NLR ≥ 4 | 0.77 (0.16–3.59) | 0.7347 |  |  |  |  |
| WBC ≥ 10500 cells/μL | 1.02 (0.20–5.14) | 0.9832 |  |  |  |  |
| Model fit |  |  |  |  |  |  |
| AUC (95% CI) | 0.901 (0.822–0.981) |  | 0.875 (0.788–0.962) |  | 0.792 (0.683–0.901) |  |
| AIC | 71.84 |  | 64.97 |  | 81.85 |  |
| Hosmer–Lemeshow test | 5.10 (df = 8) | 0.7471 | 4.63 (df = 3) | 0.2007 | 1.70 (df = 2) | 0.4273 |
| NSTEMI (n=138) | |  |  |  |  |  |
| qSOFA score ≥ 2 | 2.67 (0.09–75.59) | 0.5656 | 9.09 (0.33–247.65) | 0.1906 | 4.58 (0.18–113.87) | 0.3535 |
| Age ≥65 years | 7.10 (0.98–51.64) | 0.0529 | 11.61 (1.90–70.86) | 0.0079^*^ | 7.17 (1.57–32.80) | 0.0111 |
| MDW ≥ 20 | 27.92 (4.90–159.16) | 0.0002* | 29.76 (5.69–155.81) | <0.0001^*^ | 50.05 (10.84–231.02) | <0.0001^*^ |
| CRP ≥ 2 mg/dL | 5.21 (0.73–37.26) | 0.1001 | 9.25 (1.75–48.91) | 0.0089^*^ |  |  |
| Heart rate ≥ 90 beats/min | 0.94 (0.18–4.96) | 0.9457 |  |  |  |  |
| Respiratory rate ≥20/min | 3.76 (0.65–21.72) | 0.1395 |  |  |  |  |
| NLR ≥ 4 | 2.17 (0.35–13.61) | 0.4097 |  |  |  |  |
| WBC ≥ 10500 cells/μL | 1.84 (0.33–10.39) | 0.4918 |  |  |  |  |
| Model fit |  |  |  |  |  |  |
| AUC (95% CI) | 0.946 (0.885–1.000) |  | 0.936 (0.871–1.000) |  | 0.908 (0.819–0.996) |  |
| AIC | 62.34 |  | 58.29 |  | 63.89 |  |
| Hosmer–Lemeshow test | 13.13 (df = 7) | 0.0689 | 3.89 (df = 4) | 0.4212 | 1.02 (df = 3) | 0.7970 |

Abbreviations: AUC, area under the curve; BT, body temperature; CRP, C-reactive protein; df, degrees of freedom; NLR, neutrophil-to-lymphocyte ratio; OR, odds ratio; RR, respiratory rate; SIRS, systemic inflammatory response syndrome.

Multivariable logistic regression models were used to obtain *P* values.

^*^Statistically significant (*P* < 0.05).


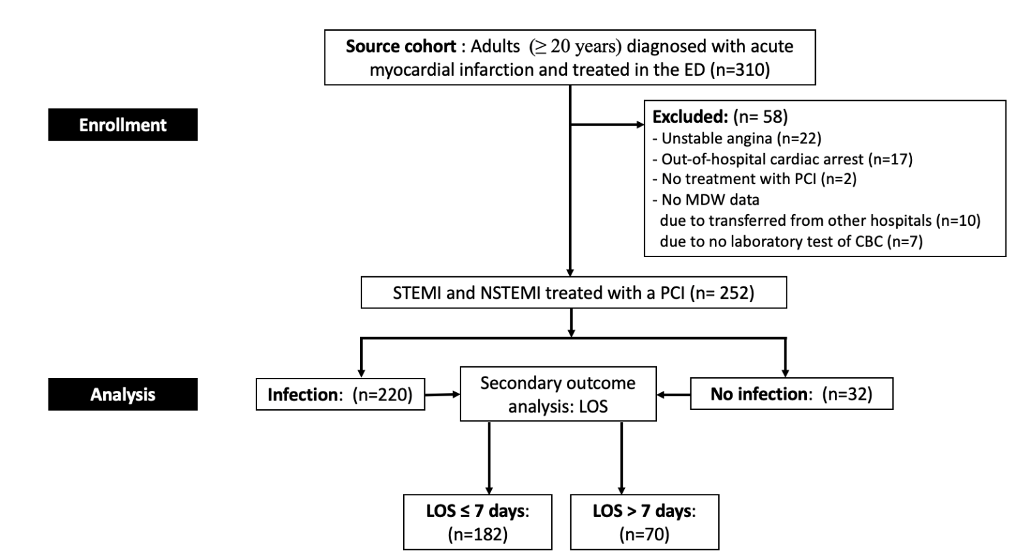


**S1 Fig. The study flow diagram.** *N* = 310 for acute coronary syndrome patients were screened for enrollment.


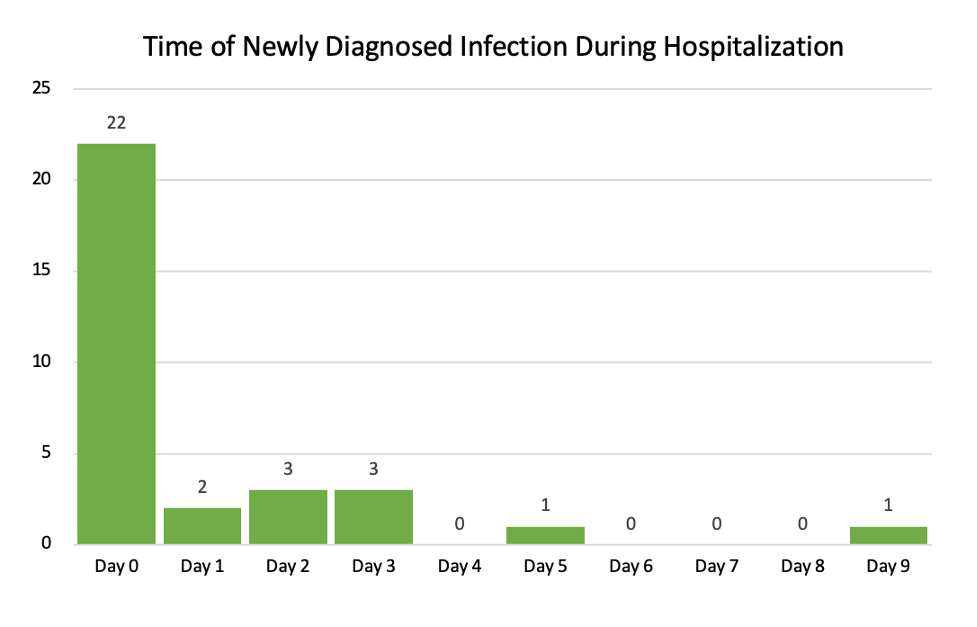


**S2 Fig. Timing of newly diagnosed infections during hospitalization.** *N* = 32 for acute coronary syndrome patients treated after PCI with infection.


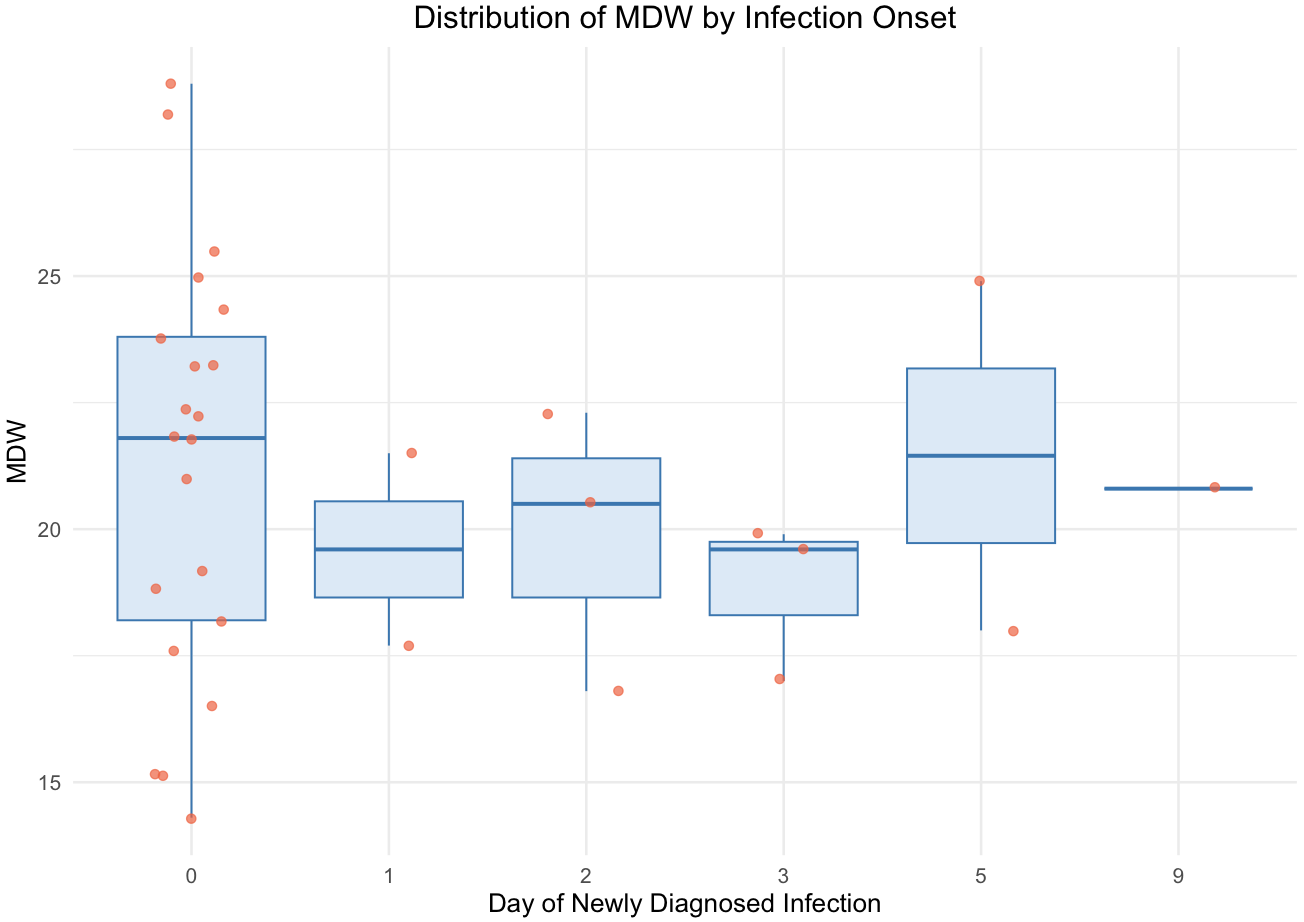


**S3 Fig. Distribution of Monocyte Distribution Width (MDW) by Infection Onset.** This box-and-whisker plot displays the distribution of MDW values across different days of infection onset, with individual data points overlaid as jittered dots. Notably, the median MDW on day 0—the day of newly diagnosed infection—was above 20, and higher than that observed on subsequent days (days 1 and 2). This finding supports our predictive model, which identified MDW ≥ 20 as a significant threshold for newly diagnosed infection, reinforcing MDW’s role as an early biomarker in acute infection settings.


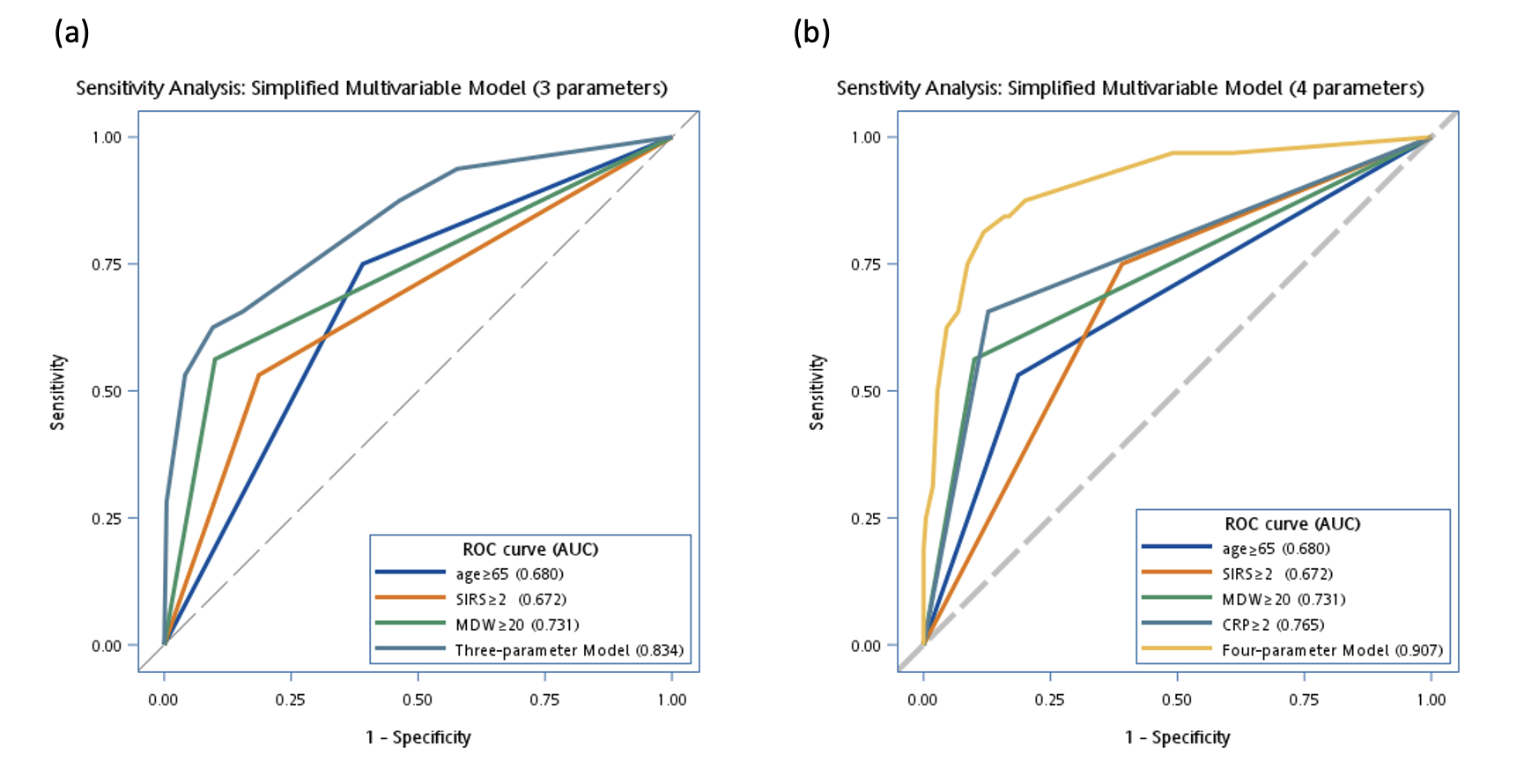


**S4 Fig. Diagnostic performance of simplified models with three parameters (a) and four parameters (b).** While the three-parameter models included age ≥65 years, MDW ≥ 20, and SIRS ≥ 2, the four-parameter models included the same three parameters in addition to CRP ≥ 2 mg/dL.


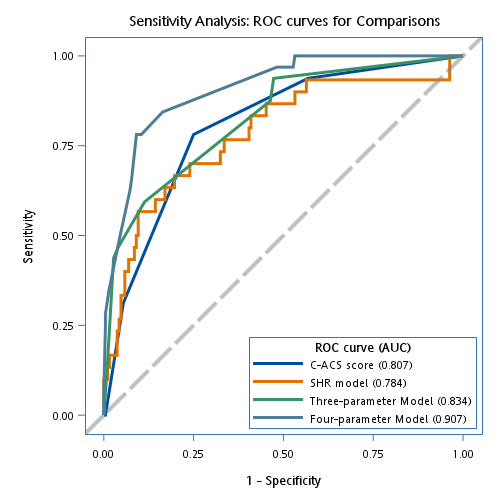


**S5 Fig. Sensitivity analysis: comparison of the diagnostic performance of the C-ACS and SHR models with our models for the prediction of infection during hospitalization.** The area under the curve (AUC) was 0.807 for the C-ACS score, 0.784 for the SHR model, 0.834 for our three-parameter model, and 0.907 for our four-parameter model.
