# Supplementary material for: Monocyte distribution width enhances the detection of infection in patients after primary percutaneous coronary intervention
Source: PLoS One. 2025 Jun 3;20(6):e0325314. doi: 10.1371/journal.pone.0325314 (PMC12132996; doi:10.1371/journal.pone.0325314)
Supplement: Attachment — Submitted filename: L-response_0506.docx [file pone.0325314.s004.docx]

We sincerely thank the reviewers for their constructive and insightful comments. In response, we have revised the manuscript accordingly to address each of the points raised. All changes made to the original manuscript have been highlighted in red text. Our detailed responses to each comment are provided below:

Responses to reviewer #1:

**(No Response)**

- We sincerely thank Reviewer #1 for taking the time to review our manuscript.

Responses to reviewer #2:

**a) On Tables 1 and 2:**

**1) Please check the SI units for the complete blood count (CBC) as follows:**

**- white blood cells (WBC), neutrophil, lymphocyte, and monocyte counts: ×10^9/L**

**- red blood cells (RBC): ×10^12/L**

**- hemoglobin (HGB): g/L**

**- hematocrit (HCT): L/L**

- We thank the reviewer for this important suggestion. We have carefully revised the units for the complete blood count (CBC) in Tables 1 and 2 as requested. Specifically, WBC, neutrophil, lymphocyte, and monocyte counts are now reported as ×10⁹/L. Corresponding adjustments have also been made in the main text to ensure consistency. (Please see the revised Table 1, Table 2, and the text on page 9.)

**2) Please delete mean CRP and NLR results because values were not-normally distributed (only median values need to be reported!). Moreover, check troponin T values distribution in the population studied (if values are not-normally distributed report median and IQ range in the text and in the Tables).**

- We appreciate the reviewer’s attention to data presentation. Following the suggestion, we have deleted the mean and standard deviation (SD) values for CRP and NLR from both the text and Tables 1 and 2.
- CRP and NLR are now reported as median and interquartile range (IQR) only.
- We reassessed the distribution of troponin-T values using the Shapiro–Wilk test, which confirmed non-normality. Accordingly, troponin-T values are now reported as median and IQR in both the main text and tables. (Please see revised Table 1, Table 2)

**b) Methods**

**Please delete the words “Using a Hematology and Clinical**

**Chemistry Analyzer” from “Biomarkers measured using a hematology and clinical chemistry analyzer” paragraph because redundant.**

- Thank you for pointing this out. We have deleted the redundant phrase “using a hematology and clinical chemistry analyzer” from the Methods section to enhance clarity.

1. **Please add to the references: Martinez-Iribarren A, Tejedor X, Sala Sanjaume A, Leis A, Dolade Botias M, Morales-Indiano C. Performance evaluation of the new hematology analyzer UniCel DxH 900. Int J Lab Hematol 2021;43:623-31 where DxH 900 analyzer showed good analytical performance in measuring MDW, the novel parameter for the early diagnosis of sepsis studied by Sheng-Feng et al.**

**c) Results**

**Edit the text on page 9 (“participant characteristics” paragraph) as follows:**

**…among all patients, the mean white blood cell (WBC) count was 18.1 ± 2.4 × 10^9/L (reference range: 4.0–10.0 ×10^9/L), the mean absolute neutrophil count was 7.48 ± 3.56 ×10^9/L (reference: 1.8–7.7 ×10^9/L), the mean lymphocyte count was 2.44 ± 1.62 ×10^9/L (reference: 1.0–3.2 ×10^9/L), the mean monocyte count was 0.77± 0.32 ×10^9/L (reference: 0.2–0.8×10^9/L),**

**and the MDW was 18.1 ± 2.4 unit (reference: <20 units). Regarding the**

**clinical chemistry tests, PCR reference value was < 5 mg/L, the**

**mean creatine kinase was 445.1 ± 878.1 unit/L (reference: 40–200 U/L), creatine kinase MB subunit was 47.3 ± 65.4 unit/L (reference: <25 U/L), and troponin-T reference value: <14 ng/L)…**

- We thank the reviewer for providing the specific suggested wording. We have edited the “Participant Characteristics” paragraph in the Results section according to the reviewer’s instructions to ensure proper unit usage, consistent format, and improved clarity.
